# Inferior vena cava tumor thrombus after partial nephrectomy for renal cell carcinoma

**DOI:** 10.1186/1756-0500-7-198

**Published:** 2014-03-29

**Authors:** Jun Akatsuka, Yasutomo Suzuki, Tsutomu Hamasaki, Takao Shindo, Masato Yanagi, Go Kimura, Yoichiro Yamamoto, Yukihiro Kondo

**Affiliations:** 1 Department of Urology, Nippon Medical School, 1-1-5 Sendagi, Bunkyo-ku, Tokyo, 113-8603, Japan; 2 Division of Diagnostic Pathology, Nippon Medical School, 1-1-5 Sendagi, Bunkyo-ku, Tokyo, 113-8603, Japan

**Keywords:** Partial nephrectomy, Renal cell carcinoma, Local recurrence, Tumor thrombus

## Abstract

**Background:**

Partial nephrectomy is now the gold standard treatment for small renal tumors. Local recurrence is a major problem after partial nephrectomy, and local recurrence in the remnant kidney after partial nephrectomy is common.

**Case presentation:**

A 77-year-old man underwent right partial nephrectomy for a T1 right renal cell carcinoma. Microscopic examination revealed a clear cell renal carcinoma, grade 2, stage pT3a. Although the surgical margin was negative, the carcinoma invaded the perirenal fat, and vascular involvement was strongly positive. Thirty months after partial nephrectomy, an enhanced computed tomographic scan showed local recurrence of the renal cell carcinoma extending into the inferior vena cava without renal mass. Hence, we performed right radical nephrectomy and intracaval thrombectomy. Microscopic examination revealed a clear cell carcinoma grade 2, stage pT3a + b. The patient is still alive with no evidence of recurrence 10 months post-procedure.

**Conclusion:**

To our knowledge, local recurrence of renal cell carcinoma extending into the inferior vena cava after partial nephrectomy has not been reported in the literature. Our case report emphasizes the importance of strict surveillance of patients after partial nephrectomy, especially for those with renal cell carcinoma positive for microvessel involvement.

## Background

During the last decade, partial nephrectomy (PN) has been accepted as an effective and safe alternative to radical nephrectomy (RN) for small renal tumors. Local recurrence in the remnant kidney after PN is common, being reported in 1%-3% of these patients.

Renal cell carcinoma (RCC) has a propensity to extend into the inferior vena cava (IVC) through the renal vein, as is seen in 4%-10% of these cases [1,2]. This involvement is usually observed at the time of initial diagnosis; local recurrence extending into the IVC after PN is exceedingly rare. We report a rare case of local recurrence of RCC extending into the IVC after PN.

## Case presentation

**Figure 1:**
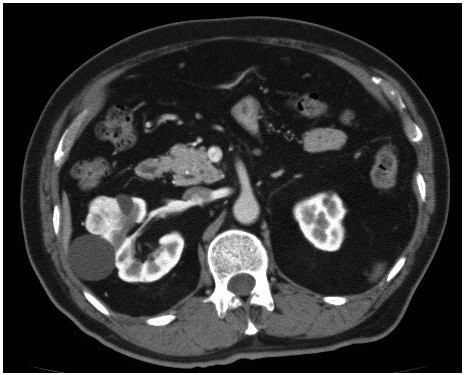
Enhanced abdominal computed tomography revealed a right renal tumor at the upper renal pole.

**Figure 2:**
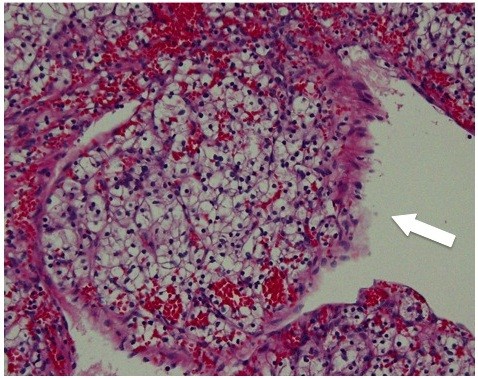
**Hematoxylin**-**eosin staining of the original partial nephrectomy specimen**, **original magnification × 400**, **demonstrated clear cell renal cell carcinoma with microvessel invasion (arrow).**

A 77-year-old man was diagnosed with a right renal tumor during a medical examination. Enhanced abdominal computed tomography (CT) revealed a right renal tumor (3.5 cm) at the upper renal pole (Figure 1). After a diagnosis of right RCC (cT1aN0M0), right PN was performed via a lateral retroperitoneal approach. Microscopic examination revealed a clear cell carcinoma, grade 2, stage pT3a. Although the surgical margin was microscopically negative, perinephric fatty tissue invasion and microvessel involvement (MVI) were positive (Figure 2). Hence, we administered combination immunotherapy (interferon (IFN)-α + meloxicam + cimetidine) as adjuvant therapy. However, this therapy was discontinued after 2 months because the patient became depressed. Because his serum creatinine level was 1.53 mg/dl, we monitored his blood test results and performed unenhanced CT of the chest and abdomen every 4 months after surgery for the first 3 years.

**Figure 3:**
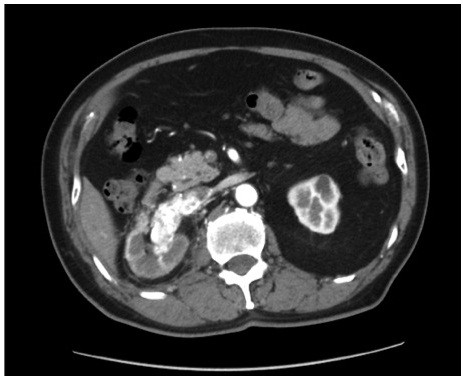
Enhanced computed tomography demonstrated an enhanced tumor thrombus in the inferior vena cava extending from the renal vein to the intrahepatic level.

**Figure 4:**
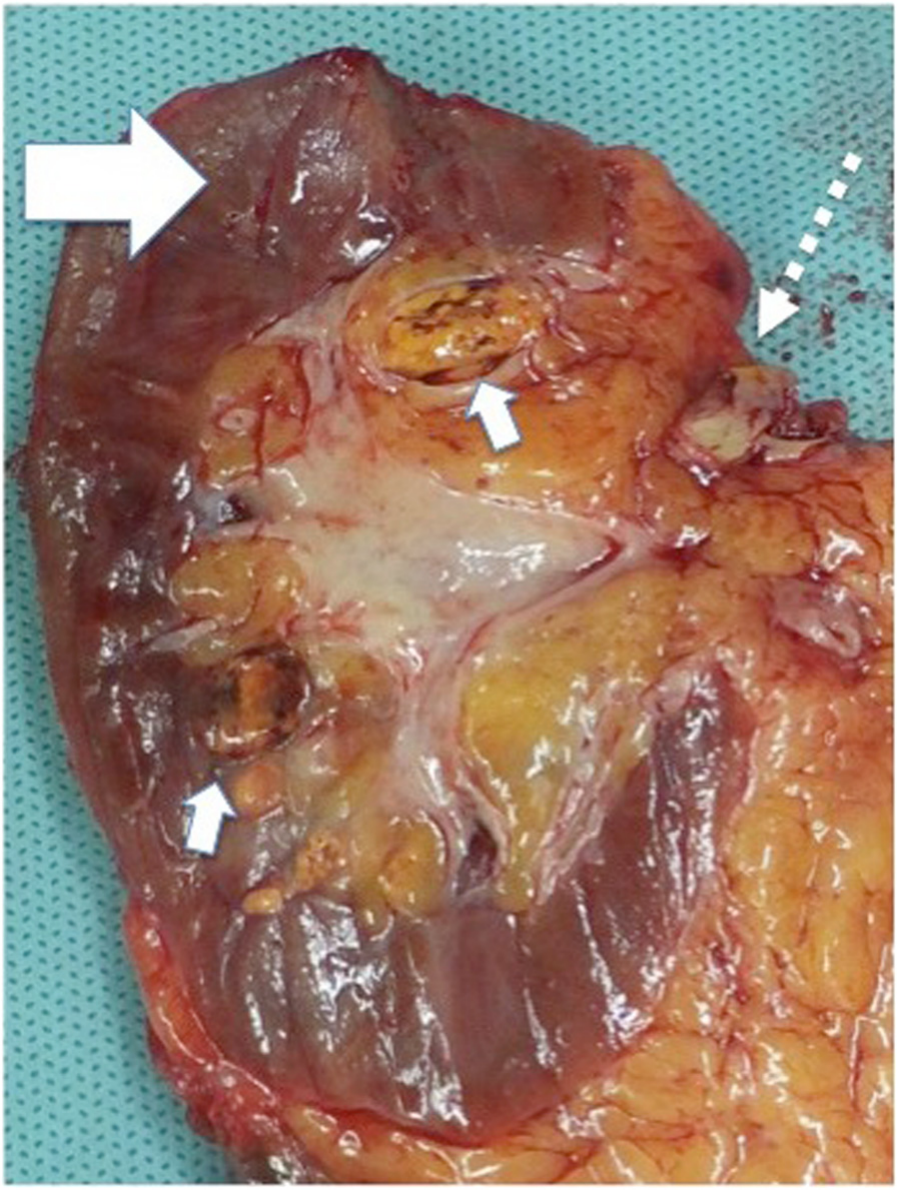
**The resected specimen included a tumor thrombus.** Large arrow: previous surgical location. Small arrows: tumor recurrence at the renal sinus. Broken arrow: tumor thrombus.

Thirty months after PN, unenhanced abdominal CT showed right renal vein swelling. Thereafter, contrast-enhanced abdominal CT confirmed the presence of a solid mass in the right renal vein extending into the IVC. The tumor extended from the renal vein to below the short hepatic vein without renal mass (Figure 3). No distant metastases were observed by brain, chest, abdominal and pelvic CT. The most feasible diagnosis at this time was local recurrence of the RCC extending into the IVC after PN (TNM staging classification: cT3bN0M0; Classification of venous tumor thrombus 4-level system: level I). We performed right RN and IVC thrombectomy using a transabdominal approach. The tumor thrombus extended from the renal vein to below the short hepatic vein and did not invade the vascular wall. Surgical time was 667 min, blood loss was 3360 ml and specimen weight was 223 g. Although there was slight recurrence of the tumor at the renal sinus, there was no recurrence at the previous surgical site (Figure 4). Microscopic examination revealed clear cell RCC, grade 2, stage pT3a + b. At 10 months after surgery, the patient is alive with no evidence of disease recurrence or metastatic disease by strict follow-up.

## Discussion

Routine medical checkups have increased the detection of small renal tumors and, hence, the necessity for PN. Several investigators have reported that PN for small renal tumors achieves equivalent oncologic outcomes and better preservation of renal function compared with RN [3,4]. Therefore, PN is now the gold standard treatment for small renal tumors.

However, local recurrence is a major problem after PN. A meta-analysis by American Urological Association Education and Research found a local recurrence rate of 2.0% after laparotomy PN, and 1.6% after laparoscopic PN [5].

Yossepowitch et al. reported clinical, pathological and follow-up data on 1,344 patients who underwent 1,390 partial nephrectomies for kidney cancer [6]. In the report, the overall 5- and 10-year rates of freedom from local disease recurrence were 97% and 93%, respectively, and the rates of freedom from metastatic progression were 96% and 93%, respectively. Patients who undergo PN need medium- and long-term follow-up, not only for the possible development of distant metastases, but also for the presence of local recurrence.

Bernhard et al. reported 26 (3.2%) ipsilateral recurrences among 809 PNs that occurred at a median time of 27 (14.5–38.2) months after PN [7]. The mean age at diagnosis was 59.3 ± 12.1 yr, and mean tumor size was 3.4 ± 1.9 cm. Twenty patients had purely local recurrence, whereas six had concurrent metastases. At the end of follow-up, 18 (69%) patients were still alive, six (23%) had died from cancer, and two (8%) had died from other causes. The risk of ipsilateral RCC recurrence after PN is significantly associated with tumor size > 4 cm, tumor bilaterality (synchronous or asynchronous), and positive surgical margin. Careful follow-up is recommended in patients presenting with such characteristics. In the present patient, the right renal tumor (3.5 cm) was removed, and the surgical margin was microscopically negative.

Systemic progression of RCC depends on tumor access to microvasculature [8]; hence, available data from several studies on this topic have indicated a significant prognostic value of MVI in such patients [9,10]. Dall’Oglio et al. demonstrated the discriminator role of MVI as a prognostic factor for RCC [11]. These investigators found that 5-year disease-free rates were 87.1% for MVI-negative cases vs. 32.6% for MVI-positive cases. In their report, MVI was the most significant predictor of RCC outcome compared with other variables such as tumor size, Fuhrman grade and sarcomatoid degeneration. In our patient, although the surgical margin was pathologically negative, MVI was positive. The site of tumor recurrence was distinct from the site of previous surgical resection. Considering the development of tumor recurrence at the renal sinus and extension into the IVC, MVI was probably responsible for the recurrence pattern in this patient. As far as we know, this is the first case of local recurrence of RCC after PN extending into the IVC. A probable cause of the rarity of advanced recurrence after PN is early discovery with strict follow-up. Our experience suggests that there might potentially be many other cases of recurrence extending into the IVC.

The National Comprehensive Cancer Network Guidelines [12] provide recommendations for follow-up after PN. Although follow-up plans should be individualized based on patient and tumor characteristics, one strategy proposes chest and abdominal imaging every 6 months for 2 years and annually for 5 years thereafter. As our patient’s renal function (BUN, 15.6 mg/ml; Cr, 1.53 mg/dl) was poor and the PN specimen was strongly positive for MVI, non-enhanced abdominal CT was performed every 4 months. Retrospective evaluation of non-enhanced abdominal CT demonstrated swelling of the right renal vein 20 months after PN. However, since the renal shape was unaltered, we could not detect local recurrence at that time. As in our patient, it is necessary to perform enhanced abdominal CT, MRI or abdominal ultrasonography after PN in order to earlier detect recurrence at the renal sinus with extension into the IVC. Despite the significant likelihood of recurrence of RCC in surgical margin- and MVI-positive cases, there is no established evidence supporting adjuvant therapy in these patients after surgery. Future clinical trials should investigate the most efficacious adjuvant strategy and agent. Our case report emphasizes the importance of pre-operative imaging which should be best performed with adequate modality and strict surveillance of patients after PN, especially for those with RCC positive for MVI.

## Conclusions

To the best of our knowledge, local recurrence extending into the IVC after PN has not previously been reported in the literature. Our case report emphasizes the importance of strict surveillance of patients after PN, especially for those with RCC positive for MVI.

## Consent

Written informed consent was obtained from the patient for publication of this Case Report and accompanying images. A copy of the written consent is available for review by the Editor-in-Chief of this journal.

## Abbreviations

PN: Partial nephrectomy; RN: Radical nephrectomy; RCC: Renal cell carcinoma; IVC: Inferior vena cava; CT: Computed tomography; MVI: Microvessel involvement.

## Competing interests

The authors declare that they have no competing interests.

## Authors’ contribution

JA wrote the manuscript and made the revisions. TH and TS obtained the consent from the patient and performed surgery on the patient. GK and YY were responsible for the histopathologic investigations and contributed to the pathological interpretation of data. MY reviewed and amended the manuscript. YS and YK were responsible for the concept, design, acquisition and interpretation of data and revision of the manuscript. All authors read and approved the final manuscript.
